# Dissociating moral identity from neural markers of the late positive potential in emotional attention to smoking and non-smoking stimuli

**DOI:** 10.3934/Neuroscience.2026006

**Published:** 2026-03-04

**Authors:** Muhammad Danial Che Ramli, Nasir Yusoff

**Affiliations:** 1 Department of Diagnostic and Allied Health Science, Faculty of Health and Life Sciences, Management and Science University, Shah Alam 40100, Selangor, Malaysia; 2 Department of Neurosciences, School of Medical Sciences, Health Campus, Universiti Sains Malaysia, Kubang Kerian 16150, Kelantan, Malaysia; 3 Brain & Behaviour Cluster, School of Medical Sciences, Universiti Sains Malaysia, Kubang Kerian 16150, Kelantan, Malaysia

**Keywords:** event-related potential, late positive potentials, attentional bias, smoking behaviour, moral identity

## Abstract

**Background:**

The influence of moral identity on smoking behaviors remains an open question, particularly among youth prone to risk-taking and moral challenges. This study examined whether moral identity modulates late positive potential (LPP) responses during emotional–cognitive processing of smoking-related and non-smoking stimuli, while hypothesizing that attentional bias operates independently of individual moral identity differences.

**Methods:**

Seventy-eight participants (M = 22 years, SD = 2.1) completed an event-related potential (ERP) session in which the LPP activity was recorded while they viewed target stimuli (1 = smoking and 2 = non-smoking) and non-target stimuli (neutral images). Prior to the ERP task, the participants completed the Moral Identity Scale (validated Malay language version) and provided sociodemographic information. The LPP components were extracted and subsequently analyzed using a mixed-design analysis of variance (ANOVA) with stimulus type (target 1 and 2, non-target) as a within-subject factor and moral identity (internalization, symbolization) as a between-subject factor.

**Results:**

Strong main effects of visual stimuli on both the LPP amplitude and latency were revealed, thus indicating robust attentional engagement with emotionally and behaviorally relevant stimuli. No interaction effects with moral identity were observed, thus suggesting that attentional mechanisms function independently of moral self-construal. Post hoc comparisons showed a consistent attentional bias toward target versus neutral stimuli, with smoking versus non-smoking differences varying across cortical regions: amplitude effects in central and temporal areas, and latency effects in parietal and occipital areas.

**Conclusion:**

Smoking-related stimuli automatically capture attention irrespective of moral identity, thus highlighting the dissociation between moral self-construal and neural markers of emotional attention.

## 1. Introduction

Moral identity—the degree to which being moral is central to one's self-concept—is a key psychological construct that helps explain why individuals choose to engage in or refrain from health-risk behaviors. It is comprised of two dimensions: internalization, which reflects the private centrality of moral traits to one's identity; and symbolization, which represents the outward expression of these traits [1]. In eastern countries such as Malaysia, cultural and religious influences amplify these dynamics. Internalization may align with religious teachings that emphasize personal responsibility for health, whereas symbolization may reflect public adherence to moral norms, social expectations, and family reputation. Recent Malaysian data suggest that both dimensions predict smoking behaviors, with gender as an additional determinant [2], although some studies indicate that moral internalization exerts a stronger influence than symbolization [3].

Understanding moral identity in the local context is particularly important because tobacco use remains one of the most preventable causes of morbidity and mortality worldwide. In Malaysia, smoking prevalence reflects strong cultural and gendered patterns: while about 41% of adult men report smoking, only about 0.5% of women do [4]. The National E-Cigarette Survey (NECS) 2020 reported measurable prevalence of e-cigarette and vape use among Malaysian adults [5]. These patterns highlight the urgent need to identify psychological and neural mechanisms that underlie smoking behaviors, especially in cultural settings where health decisions are embedded in moral, religious, and social norms. In collectivist societies such as Malaysia, identifying as a non-smoker may represent not only a health-related choice but also a moral stance tied to family duty, religious adherence, and social responsibility [6]–[8].

From a neuroscience perspective, smoking-related cues elicit strong affective and attentional responses. These responses are often measured using event-related potentials (ERPs), particularly the Late Positive Potential (LPP), which reflects sustained motivated attention to emotionally or motivationally relevant stimuli [9]–[11]. ERP research consistently shows that the LPP is sensitive to smoking images [12]–[15], and larger LPP amplitudes reflect greater attentional and emotional engagement with salient drug cues, which is theorized to contribute to craving and increased relapse risk [16]. However, the relationship between LPP modulation and moral identity remains largely unexplored. While some studies suggest that individuals with a stronger moral identity demonstrate an enhanced regulation of responses to risky or temptation-related cues [17], others find little to no relationship [18].

A notable limitation of the current literature is its Western bias. Most ERP studies on smoking cues were conducted in individualistic cultural contexts [19],[20], where moral reasoning is framed around personal autonomy and individual choices. In contrast, Malaysia represents a collectivist, multi-religious society in which moral values are deeply embedded in cultural traditions and religious norms. For example, Islamic rulings (fatwa) which declare smoking as prohibited emphasize both the health consequences and the moral obligation to protect oneself and others [21]. These cultural and religious contexts offer a unique lens to examine how moral identity may shape neural responses to smoking cues.

Thus, these gaps underscore the need for an exploratory investigation into whether moral identity modulates the LPP reactivity to smoking-related stimuli in a Malaysian population. Clarifying whether these constructs are linked—or whether they operate independently—can guide future research. If preliminary patterns suggest an association, then culturally grounded moral values could be incorporated into later intervention works. If no link emerges, then future studies may explore how moral identity interacts with other mechanisms, such as cue reactivity or emotion regulation. Adopting a cultural neuroscience perspective ensures that models of self-regulation remain sensitive to local moral norms and societal expectations surrounding smoking.

## 2. Materials and methods

### 2.1. Research design and participants

This laboratory-based observational study was conducted at the Neuroscience Laboratory, Hospital Universiti Sains Malaysia, using the ERP technique. The sample is comprised of 78 young adults (mean age = 22 ± 2.1 years), with more than half being male participants (58%, N = 45). They represented diverse ethnic backgrounds (74% Malay, 19% Chinese, 5% Indian) and religious affiliations (73% Muslim), and resided in Kota Bharu city, Kelantan, Malaysia. The educational levels included undergraduates (82%), postgraduates (12%), and diploma or high school certificate holders (6%). Most participants were lifelong non-smokers (90%), followed by current smokers (6%) and former smokers (4%). The participants met the inclusion criteria of being right-handed, free from neuropsychiatric or chronic medical conditions, having normal or corrected-to-normal vision, and no history of serious health risk behaviors (e.g., drug abuse, alcohol dependence, unprotected sex). Thirty-eight percent used corrective glasses. Recruitment employed convenience sampling via social media. Written informed consent was obtained, and the study was approved by the Universiti Sains Malaysia Human Ethics Committee (USM/JEPeM/20060297).

### 2.2. Moral identity assessment

The participant's moral identity patterns—high internalization–low symbolization, low internalization–high symbolization, high internalization–high symbolization, and low internalization-low symbolization group—were assessed using the validated Malay Version of the Moral Identity Scale (MIS) [22] alongside sociodemographic data. The MIS measures internalization (intrinsic importance of moral traits) and symbolization (external expression) via 3 and 5 items, respectively, rated on a 7-point Likert scale after visualizing positive traits (e.g., caring, fair, honest). Two low-loading symbolization items from the original version (<0.5) were excluded.

The scores were categorized using the median split method [23]: internalization (median = 16 (range: 11–21), ≥16 = high, <16 = low) and symbolization (median = 24 (range: 15–30), ≥24 = high, <24 = low). All statistical assumptions were met.

### 2.3. ERP recording and analysis

**Figure 1.**
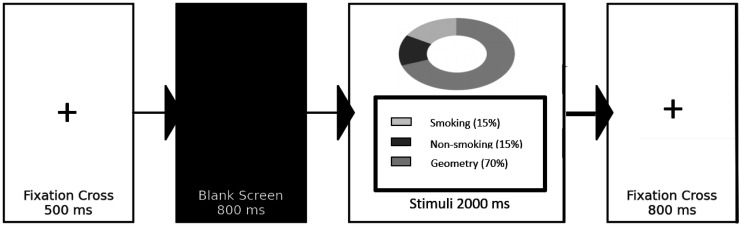
Event Related Potential paradigm. Each trial included fixation (500 ms), blank screen (800 ms), and stimulus presentation (2000 ms; smoking 15%, non-smoking 15%, geometric 70%), followed by fixation (800 ms). ERPs were time-locked to the stimulus onset for late positive potential (LPP) analysis.

The participants viewed 200 randomly presented trials of smoking-related, non-smoking-related, and neutral (geometrical) images (ratio 1:1:3; 30% target, 70% non-target) in an oddball paradigm (Figure 1). The oddball paradigm is an experimental design in ERP research where infrequent stimuli (target) are embedded within frequent standard stimuli (non-target) to measure brain responses related to novelty detection, attention, and cognitive processing. Each trial began with a 500 ms fixation cross, followed by an 800 ms blank screen and a 2000 ms image presentation. In this ERP paradigm, the two fixation crosses served distinct functions. The first fixation cross (500 ms) acted as a pre-stimulus baseline period, ensured that the participant's attention was centrally focused, and allowed the electroencephalogram (EEG) signal to stabilize before stimulus onset. This provided a consistent baseline to average the ERP components. The second fixation cross (800 ms, presented in black) appeared after the stimulus and functioned as an inter-stimulus interval (ISI). Its longer duration helped minimize the carryover effects from the preceding trial, allowed cognitive and emotional processing to return toward baseline, and reduced the anticipatory responses before the next stimulus was presented, which was particularly important to analyze the late components such as the LPP. Stimuli were displayed on a computer screen connected to a NetAmps 300 amplifier, with the EEG recorded using a 128-channel HydroCel Geodesic Sensor Net.

Images were validated by three public health experts using the Content Validity Index (CVI) [24]. A total of 20 images were selected, 10 depicting health-risk smoking behavior and 10 depicting non-smoking behavior (i.e., healthy sports activities), all of which achieved an item-level CVI (I-CVI) of 1 and were standardized for brightness and size.

The EEG signals were sampled at 500 Hz, filtered (0.3–30 Hz), segmented from −200 to +1000 ms relative to stimulus onset, and baseline-corrected. The LPP was quantified by extracting the mean amplitude within the 600–900 ms post-stimulus window, whereas the peak latency values were identified using automated peak detection across the full ERP waveform, which is not limited to this amplitude window. Brain potential data (amplitude and latency) were recorded from five scalp regions using the international 10/20 system with Ag/AgCl electrodes. The regions included fronto-parietal (Fp1, Fp2), frontal (F3, F4, F7, F8, Fz), central (C3, C4, Cz), temporal (T3, T4, T5, T6), and occipital sites (O1, O2). Electrodes across the fronto-parietal, frontal, central, temporal, and occipital regions were included to provide a broad explanatory coverage of neural processes involved in visual perception, emotional evaluation, and attentional engagement with smoking cues. Additionally, this montage ensured adequate sampling of centro-parietal sites where the LPP is typically maximal, thus allowing a reliable extraction of the component. The amplitude of the LPP reflects the intensity of emotional processing and motivational relevance of a stimulus. Meanwhile, the latency of the LPP reflects the timing of emotional or motivational processing—essentially when the brain begins generating the sustained positive deflection. Artefacts (e.g., ocular) were removed, bad channels were identified and interpolated, data were re-referenced and epoched, and the resulting epochs were averaged to produce ERPs after mapping data to the international 10–20 montage. Analyses of the LPP amplitude and latency were performed in SPSS, v27, using mixed-design analyses of variance (ANOVAs)—one model for amplitude and another for latency. In both models, the four categories of moral identity served as the between-subjects factor, and the three categories of visual stimuli served as the within-subjects factor. The Huynh–Feldt correction was applied when the assumption of sphericity was violated. Post hoc pairwise comparisons using the Bonferroni correction were conducted to further clarify the pattern of differences between the categories.

## 3. Results

### 3.1. LPP amplitude and latency

The two-way ANOVA revealed a robust main effect of visual stimuli on the LPP amplitudes across all cortical regions, irrespective of moral identity traits. Significant differences were observed in the frontoparietal [*F*(1.8, 131.4) = 16.9, *p* < 0.001], frontal [*F*(2, 73) = 5.3, *p* < 0.01], central [*F*(1.64, 119.4) = 39.99, *p* < 0.001], temporal [*F*(2, 73) = 99.0, *p* < 0.001], parietal [*F*(2, 145) = 63.8, *p* < 0.001], and occipital [*F*(2, 73) = 53.4, *p* < 0.001] regions (see Table 1).

A parallel pattern emerged for the LPP latency. Except for the central region [*F*(2, 73) = 1.9, n.s.], all cortical areas demonstrated a significant main effect of visual stimuli. Significant effects were found in the frontoparietal [*F*(1.8, 134.5) = 16.9, *p* < 0.001], frontal [*F*(1.7, 119.6) = 45.6, *p* < 0.001], temporal [*F*(1.8, 138.8) = 1.8, *p* < 0.001], parietal [*F*(1.8, 133.6) = 72.0, *p* < 0.001], and occipital [*F*(1.6, 119.8) = 104.9, *p* < 0.001] regions (see Table 2).

In contrast, the interaction between moral identity and emotional processing was nonsignificant for both amplitude and latency across all brain regions. For amplitude, nonsignificant interactions were found in the frontoparietal [*F*(5.3, 131.4) = 0.74, *p* = 0.51], frontal [*F*(6, 148) = 1.0, *p* = 0.24], central [*F*(4.9, 119.4) = 1.3, *p* = 0.54], temporal [*F*(6, 148) = 0.33, *p* = 0.72], parietal [*F*(5.9, 145) = 1.6, *p* = 0.71], and occipital [*F*(6, 148) = 0.6, *p* = 0.77] regions. Similarly, latency analyses showed no significant interactions for the frontoparietal [*F*(5.5, 134.5) = 0.7, *p* = 0.62], frontal [*F*(5.1, 126.9) = 0.56, *p* = 0.74], central [*F*(6, 148) = 0.64, n.s.], temporal [*F*(1.8, 138.8) = 0.39, *p* = 0.66], parietal [*F*(5.4, 133.6) = 0.6, *p* = 0.68], and occipital [*F*(4.9, 119.8) = 0.43, *p* = 0.82] regions (see Table 1 and 2).

These findings demonstrate that while the emotional significance of visual stimuli robustly modulated both the magnitude (amplitude) and timing (latency) of neural responses, these effects were not further differentiated by individual differences in moral identity.

### 3.2. Pairwise comparisons

Post hoc pairwise comparisons (Bonferroni corrected) were conducted to further specify the differences between the stimuli categories.

For amplitude, two main findings emerged (see Table 3). First, a clear attentional bias was observed when comparing the target images (smoking and non-smoking) with the non-target neutral images. This effect was consistent across nearly all brain regions, particularly the fronto-parietal, frontal, temporal, parietal, and occipital areas, with the central region being the notable exception. Second, a direct comparison between the two categories of target images revealed significant differences: smoking-related images elicited stronger amplitudes than non-smoking images in the central region (*p* < 0.001), with a smaller but still significant effect in the temporal region (*p* < 0.05).

For latency, a comparable but distinct pattern was found (see Table 4). Target images, both smoking and non-smoking, were more rapidly processed than non-target neutral images across nearly all regions, again with the central region showing no significant differences. The strongest latency effects were evident in the fronto-parietal, frontal, temporal, parietal, and occipital areas. Moreover, distinctions between smoking and non-smoking stimuli emerged primarily in the parietal and occipital regions, where the non-smoking images required longer cognitive processing than the smoking images. This finding aligns with the hypothesis that the smoking-related images are processed more quickly than the non-smoking images.

**Table 1.** LPP amplitude across brain regions: influence of moral identity on stimulus type.

Brain Region	Mean (Standard Deviation)^1^												F^a^/F^b^	df^a/^/df^b^
	Smoking				Non-smoking				Neutral					
	HiLs	LiHs	HiHs	LiLs	HiLs	LiHs	HiHs	LiLs	HiLs	LiHs	HiHs	LiLs		
Frontoparietal^a^**^/b (ns)^	25.7 (16.7)	20.8 (15.7)	19.6 (14.0)	20.7 (16.4)	16.9 (9.7)	20.2 (12.3)	17.7 (9.2)	18.5 (11.4)	13.9 (6.1)	13.5 (5.9)	9.9 (4.8)	13.0 (5.9)	16.9^+^/ 0.7^+^	1.8^+^/ 5.3^+^
Frontal^a^**^/b (ns)^	23.3 (11.3)	20.8 (9.8)	25.8 (10.1)	21.5 (11.7)	19.8 (8.4)	21.6 (6.7)	22.1 (9.8)	18.9 (9.3)	18.7 (7.3)	19.8 (8.1)	16.7 (7.8)	18.8 (8.5)	5.3^++^/ 1.0^++^	2^++^/ 6^++^
Central^a^***^/b (ns)^	10.4 (6.1)	9.9 (5.5)	13.3 (8.1)	11.1 (6.1)	6.1 (2.2)	6.8 (3.6)	6.6 (3.0)	7.3 (3.4)	6.8 (3.6)	6.0 (2.1)	5.7 (3.5)	5.9 (2.3)	40.1^+^/ 1.3^+^	1.6^+^/ 4.9^+^
Temporal^a^***^/b (ns)^	9.7 (3.7)	10.7 (4.3)	10.6 (4.9)	10.9 (4.1)	12.1 (4.6)	11.6 (3.5)	11.6 (4.9)	12.1 (3.5)	6.0 (1.5)	5.6 (1.4)	5.6 (1.7)	6.1 (1.0)	99.0^++^/ 0.3^+=^	2^++^/ 6^++^
Parietal^a^***^/b (ns)^	11.5 (5.1)	12.4 (8.3)	11.3 (6.1)	12.6 (8.4)	15.4 (6.8)	16.3 (6.1)	9.8 (5.1)	13.8 (7.6)	5.9 (3.7)	6.4 (4.4)	4.7 (3.8)	5.3 (4.2)	63.8^+^/ 1.6^+^	2^+^/ 5.9^+^
Occipital^a^***^/b (ns)^	6.4 (3.7)	6.5 (3.4)	5.9 (3.9)	6.8 (4.1)	6.5 (2.6)	7.3 (3.3)	5.7 (3.5)	6.7 (3.3)	3.7 (1.5)	2.9 (1.2)	2.9 (1.9)	3.0 (1.5)	53.4^++^/ 0.6^++^	2^++^/ 6^++^

Note: HiLs = High internalization Low symbolization; LiHs = Low internalization High symbolization; HiHs = High internalization High symbolization; LiLs = Low internalization Low symbolization; a = main effect; b = interaction effect; ns = not significant; ^1^Microvolt-µV; ^+^Huynh Felt result (Sphericity Mauchly, p < 0.05); ^++^Multivariate Pillai's Trace (Sphericity Mauchly, *p* > 0.05); *p < 0.05; **p < 0.01; ***p < 0.001.

**Table 2.** LPP latency across brain regions: influence of moral identity on stimulus type.

Brain Region	Mean (Standard Deviation)^1^												F^a^/F^b^	df^a/^/df^b^
	Smoking				Non-smoking				Neutral					
	HiLs	LiHs	HiHs	LiLs	HiLs	LiHs	HiHs	LiLs	HiLs	LiHs	HiHs	LiLs		
Fronto-Parietal^a^***^/b (ns)^	392.8 (44.8)	388.4 (47.2)	402.4 (47.6)	387.9 (45.7)	390.9 (48.9)	395.5 (47.3)	396.7 (44.5)	387.0 (51.0)	364.0 (47.6)	372.6 (45.0)	348.8 (51.5)	351.5 (44.8)	16.8^+^/ 0.7^+^	1.8/ 5.5^+^
Frontal^a^***^/b (ns)^	1003.7 (62.4)	1006.5 (55.8)	1011.6 (72.9)	1011.5 (82.9)	1016.2 (66.6)	1005.8 (56.6)	1019.4 (51.8)	1009.1 (60.8)	925.9 (85.8)	923.6 (99.1)	906.4 (85.7)	897.9 (91.8)	45.6^+^/ 0.6^+^	1.7^+^/ 5.1^+^
Central^a (ns)/b (ns)^	533.4 (46.1)	555.3 (46.0)	530.0 (52.9)	532.4 (55.1)	553.5 (51.0)	565.9 (53.5)	531.0 (51.5)	556.6 (57.0)	552.8 (61.2)	547.0 (51.4)	529.6 (65.0)	562.5 (59.2)	1.9^++^/ 0.6^++^	2.0^++^/ 6.0^++^
Temporal ^a (ns)/b (ns)^	686.2 (60.5)	730.6 (45.6)	697.1 (49.6)	690.5 (45.2)	683.5 (44.3)	724.6 (48.5)	725.2 (62.5)	702.8 (43.5)	743.1 (74.0)	757.0 (60.0)	759.1 (64.5)	762.4 (64.9)	25.7^+^/ 0.39^+^	1.8^+^/ 1.8^+^
Parietal ^a^***^/b (ns)^	482.7 (34.4)	491.0 (25.6)	476.1 (29.0)	494.0 (38.6)	493.0 (45.8)	524.4 (62.2)	499.3 (55.6)	500.3 (56.1)	562.4 (66.0)	568.6 (58.9)	570.0 (53.1)	571.5 (59.6)	72.0^+^/ 0.6^+^	1.8^+^/ 5.4^+^
Occipital ^a^***^/b (ns)^	318.3 (14.2)	307.9 (13.8)	309.5 (11.1)	311.5 (12.7)	325.7 (30.6)	339.5 (42.0)	332.9 (40.8)	322.5 (27.2)	374.1 (49.2)	385.9 (45.6)	389.7 (41.5)	384.2 (43.8)	104.9^+^/ 0.4^+^	1.6^++^/ 4.9^++^

Note: HiLs = High internalization, Low symbolization; LiHs = Low internalization, High symbolization; HiHs = High internalization, High symbolization; LiLs = Low internalization, Low symbolization; a = main effect; b = interaction effect; ns = not significant; ^1^Miliseconds; ^+^Huynh Felt result (Sphericity Mauchly, *p* < 0.05); ^++^Multivariate Pillai's Trace (Sphericity Mauchly, *p* > 0.05); *p < 0.05; **p < 0.01; ***p < 0.001.

**Table 3.** Pairwise comparisons of LPP amplitudes (*microvolt*) across smoking, non-smoking and neutral stimuli.

Brain Region	Mean Difference	Std. Error	95% CI (lower to upper)
Fronto-parietal
1 vs. 2	3.4	1.8	neg1.0–7.7
1 vs. 3***	9.1	1.7	4.9–13.2
2 vs. 3***	5.7	1.2	2.8–8.5
Frontal
1 vs. 2	2.2	1.2	neg0.7–5.1
1 vs. 3**	4.1	1.4	0.8–7.5
2 vs. 3	2.0	1.2	neg1.0–5.0
Central
1 vs. 2***	4.4	0.7	2.8–6.1
1 vs. 3	5.0	0.7	3.2–6.8
2 vs. 3	0.6	0.4	neg0.5–1.6
Temporal
1 vs. 2*	neg1.4	0.5	neg2.5–neg0.12
1 vs. 3***	4.7	0.5	3.5–5.8
2 vs. 3***	6.0	0.4	5.0–7.1
Parietal
1 vs. 2	neg2.0	0.9	neg4.1–0.2
1 vs. 3***	6.4	0.7	4.6–8.2
2 vs. 3***	8.4	0.7	6.7–10.1
Occipital
1 vs. 2	neg0.1	0.5	neg1.3–1.0
1 vs. 3***	3.2	0.4	2.3–4.3
2 vs. 3***	3.4	0.4	2.5–4.4

Note: 1: Smoking behavior image (TARGET 1); 2: Non-smoking behavior image (TARGET 2); 3: Geometry-neutral image (NON-TARGET); *p < 0.05; **p < 0.01; ***p < 0.001; neg = negative.

**Table 4.** Pairwise comparisons of LPP latency (milliseconds) across smoking, non-smoking and neutral stimuli.

Brain Region	Mean Difference	Std. Error	95% CI (Lower to upper)
Fronto-parietal
1 vs. 2	0.03	5.1	neg12.4–12.4
1 vs. 3***	32.7	7.6	14.2–51.2
2 vs. 3***	32.6	6.9	15.7–49.5
Frontal
1 vs. 2	3.6	8.8	neg18.0–25.1
1 vs. 3***	91.5	13.6	58.1–124.9
2 vs. 3***	87.9	12.0	58.5–117.4
Central
1 vs. 2	neg14.4	7.2	neg32.1–3.2
1 vs. 3	neg10.4	8.6	neg31.5–10.7
2 vs. 3	4.0	8.8	neg17.6–25.7
Temporal
1 vs. 2	neg7.0	6.4	neg22.7–8.7
1 vs. 3***	neg53.7	8.8	neg72.5–neg32.1
2 vs. 3***	neg46.7	8.7	neg67.9–neg25.4
Parietal
1 vs. 2**	neg18.3	5.8	neg32.6–neg4.1
1 vs. 3***	neg81.7	7.0	neg98.8–neg64.6
2 vs. 3***	neg63.4	8.4	neg83.9–neg42.9
Occipital
1 vs. 2***	neg18.6	3.8	neg27.8–neg9.4
1 vs. 3***	neg71.7	5.0	neg83.9–neg59.5
2 vs. 3***	neg53.1	6.2	neg68.4–neg37.9

Note: 1: Smoking behavior image (TARGET 1); 2: Non-smoking behavior image (TARGET 2); 3: Geometry-neutral image (NON-TARGET); *p < 0.05; **p < 0.01; ***p < 0.001; neg = negative.

## 4. Discussion

The present study examined how emotionally salient visual stimuli—smoking, non-smoking, and neutral images—modulate neural processing as indexed by the LPP amplitude and latency, and whether these effects are influenced by individual differences in moral identity. Three important findings emerged:

- Strong main effects of visual stimuli on both the LPP amplitude and latency across most cortical regions indicate robust attentional engagement with emotionally or behaviorally relevant stimuli.
- No interaction effects with moral identity were observed, thus suggesting that these attentional mechanisms largely operate independently of moral self-construal.
- Post hoc comparisons revealed a consistent attentional bias toward target stimuli versus neutral ones for both amplitude and latency. In contrast, differences between the smoking and non-smoking targets were measure-specific: amplitude effects emerged in central and temporal regions, while latency effects appeared in parietal and occipital regions.

Based on these findings, several important points can be highlighted. First, the emotional significance was reflected in both the strength and speed of the neural responses. Our results replicate and extend the well-established pattern that emotionally arousing stimuli amplify the LPP amplitude compared to neutral cues [25]. Beyond basic emotional processing, recent evidence further demonstrated that the LPP is closely tied to psychopathology. For example, one study showed that anxiety sensitivity and intolerance of uncertainty uniquely explain the relationship between LPP responses to negative stimuli and generalized anxiety disorder symptoms [26]. Importantly, we also observed a significant reduction in the LPP latency (specifically for the parietal and occipital areas), thus suggesting faster neural engagement for smoking image processing, which is a critical complement to amplitude measures.

However, the brain regions implicated in amplitude and latency effects differed. Several ERP studies provided support for amplitude effects in central and temporal regions in response to smoking-related stimuli. For example, Versace et al. [27] recorded dense-array ERPs in 116 smokers that viewed neutral, emotional, and cigarette-related pictures; they found significantly greater positivity (LPP) for cigarette cues over central, parietal, and frontal sites in the 452–508 ms window, similar to the LPP elicited by emotional stimuli. Franken et al. [28] used a conditioned learning paradigm and reported larger P3 and LPP amplitudes over a centro-frontally distributed region, thus suggesting that smoking cues engage motivated attention systems in smokers. In addition, a large ERP sample of smokers, cigarette-related cues elicited significantly larger late positive potentials over central-parietal electrode sites than neutral images, supporting the interpretation that these central regions reflect the motivational salience of smoking cues [29]. These findings collectively support the notion that central (and to some degree temporal/parietal) brain regions are engaged by smoking-related cues, which is consistent with the idea that these areas support higher-order attentional control and the elaboration of motivationally relevant stimuli.

Some earlier ERP research supports the notion that affective stimuli elicit late positive potentials whose generation involves posterior brain regions (parietal/occipital), which is consistent with changes in perceptual and attentional mechanisms. For instance, Keil et al. [30] used a 129-sensor EEG to show that the slow-wave (LPP-like) modulation for emotional vs neutral pictures arises from sources in occipital and posterior parietal cortices. Schupp et al. [31] briefly presented affective pictures (120 ms) and demonstrated larger LPPs over centro-parietal sites, plus early negative shifts over temporo-occipital sensors, thus suggesting rapid perceptual tuning to emotional content. Cuthbert et al. [32] reported a sustained, slow positive potential (LPP) that started ~200–300 ms and lasted for up to a second over parietal/occipital regions for emotionally arousing pictures, thus supporting the role of the posterior cortex in sustained emotional processing.

Although the LPP has traditionally been interpreted as reflecting automatic, bottom-up attentional engagement with emotionally salient stimuli, more recent research indicates that the functional meaning of the LPP can vary across different scalp regions. For example, Bonilla-Santos et al. [33] examined emotional processing in children involved in bullying using ERP measures. The victims showed notably higher LPP amplitudes, especially in anterior, posterior, and central regions, thus indicating heightened neural reactivity, even to neutral social images, which suggests an increased sensitivity in emotional processing. Similarly, Klein et al. [34] found that individuals with PTSD exhibited heightened early LPP amplitudes in response to socially threatening stimuli. Additionally, they observed region-specific effects: the anterior LPP activity was greatest for positive words, whereas socially and physically threatening words produced the strongest modulation in posterior LPP regions. These regional distinctions were acknowledged, noting that the topographical distribution of the LPP may help clarify whether the observed effects relate more closely to perceptual-attentional processes (posterior regions) or higher-order evaluative and regulatory processes (anterior regions). Recognizing these functional differences strengthens the interpretation of current study results and aligns with current advancements in affective neuroscience.

This dual evidence strongly supports the notion that the LPP captures both the intensity and the temporal dynamics of emotional processing. Extending this perspective, recent work demonstrates that the LPP not only indexes emotional significance but also tracks the allocation of attentional and affective resources to socially meaningful information. For instance, enhanced parietal LPP amplitudes during self-relative compared with other-referential processing in children indicate preferential neural engagement with self-relevant cues [35]. This aligns with our findings by further highlighting the functional relevance of LPP amplitude, especially in emotionally laden contexts like smoking cues.

Second, moral identity did not modulate the LPP, thus suggesting that bottom-up emotional processing dominates over the trait-based variability. Despite growing interest in moral identity's role in social cognition, our findings suggest that LPP responses to emotional stimuli are largely driven by the stimulus properties, not by individual differences in moral self-conception. This aligns with the notion that early neural responses, such as the LPP, reflect automatic, bottom-up attentional processes. Nonetheless, some recent evidence contradicts this. For instance, Jian et al. found that in adolescents with hearing loss, the LPP was modulated by moral priming [36]. Furthermore, Pletti et al. showed that moral identity influenced earlier components (EPN, N2) during moral evaluations, though the effect on the LPP was more subtle [37]. Together, these findings suggest that moral identity may affect earlier, more controlled processing stages rather than the stimulus-driven LPP window.

Third, there is consistency—though differential across measures—in the attentional bias. Pairwise comparisons between target and neutral stimuli underscore a consistent attentional bias across both the amplitude and latency domains, thus reinforcing the notion of motivational capture by relevant cues regardless of moral identity. This echoes the cue reactivity literature. Notably, smoking versus non-smoking stimuli diverged across measures: amplitude effects emerged in the central and temporal regions, whereas latency differences appeared in the parietal and occipital regions.

A similar principle emerges in broader contexts, such as moral decision-making with autonomous vehicles. In an ERP study that extended the Moral Machine paradigm, the participants observed AI-driven traffic decisions, with incongruent choices eliciting increased P3 and LPP amplitudes [38]. Across both domains, ERPs provided converging evidence that the stimulus significance—whether health-related or socially moral—is encoded through distinct temporal and spatial dynamics.

Moreover, an ERP study showed that both early components (N200) and LPP during visual processing were modulated by subjective preference, with larger LPP amplitudes reflecting greater emotional engagement [39]. While much of the evidence emphasizes bottom-up influences, research by Littel and Franken showed that cognitive regulation strategies can modulate LPP responses to smoking cues [40]. Although our study did not incorporate regulation conditions, these insights highlight a promising avenue for future research.

This study advances the literature in several important ways. First, it demonstrates that the LPP latency, alongside amplitude, is sensitive to emotionally salient stimuli, thus highlighting an underutilized yet valuable measure in ERP research. Second, it provides evidence that moral identity may not significantly influence neural responses within the LPP window, thus suggesting instead that moral self-concept may exert its effects either earlier or at later, decision-related stages. Third, by showing that amplitude and latency measures yield complementary insights, the study methodologically contributes to a more nuanced understanding of attentional bias.

Despite its strengths, several limitations warrant consideration. First, the absence of a cognitive regulation manipulation limits the ability to determine whether moral identity might modulate the top-down control of LPP responses—an important direction given prior evidence [40]. Second, the cultural and demographic specificity of our sample may shape the salience of smoking-related images; thus, future comparative work would be valuable. Third, the inclusion of earlier ERP components, such as the EPN and N2, could further clarify whether moral identity exerts more subtle influences before the LPP window [37].

## 5. Conclusion

Emotionally salient smoking-related cues automatically capture attention, independent of moral identity traits. This suggests that the reliance on moral or value-based appeals alone may be insufficient to counteract the powerful bottom-up processes that sustain smoking behaviors. For public policy and population health, the results highlight the need for evidence-based strategies that directly address neural mechanisms of attentional bias, such as restricting exposure to smoking cues through advertising bans, enhancing warning labels, and integrating cognitive training into cessation programs. Targeting these automatic processes can strengthen preventive policies and improve population-level health outcomes.

## Use of AI tools declaration

The authors declare they have not used Artificial Intelligence (AI) tools in the creation of this article.
